# Facilitating and barrier factors to the implementation of a transitional care program: a qualitative study of hospital coordinators in South Korea

**DOI:** 10.1186/s12913-024-10720-x

**Published:** 2024-02-23

**Authors:** Yukyung Park, Su Mi Jung, Su Kyoung Kim, Heui Sug Jo

**Affiliations:** 1 Department of Preventive Medicine, Kangwon National University Hospital, Chuncheon, Republic of Korea; 2 Team of Public Medical Policy Development, Gangwon State Research Institute for People’s Health, Chuncheon, Republic of Korea; 3 Department of Health Policy and Management, School of Medicine, Kangwon National University, Chuncheon, Republic of Korea

**Keywords:** Transitional Care Service, Implementation factor, Coordinators’ perspective, Institutionalization

## Abstract

**Background:**

Transitional care is an integrated service to ensure coordination and continuity of patients’ healthcare. Many models are being developed and implemented for this care. This study aims to identify the facilitators and obstacles of project performance through the experiences of the coordinator in charge of the Community Linkage Program for Discharge Patients (CLDP), a representative transitional care program in Korea.

**Method:**

Forty-one coordinators (nurses and social workers) from 21 hospitals were interviewed using a semi-structured questionnaire, and thematic analysis was performed.

**Result:**

Three themes were found as factors that facilitate or hinder CLDP: Formation and maintenance of cooperative relationships; Communication and information sharing system for patient care; and interaction among program, regional, and individual capabilities. These themes were similar regardless of the size of the hospitals.

**Conclusion:**

A well-implemented transitional care model requires a program to prevent duplication and form a cooperative relationship, common computing platform to share patient information between institutions, and institutional assistance to set long-term directions focused on patient needs and support coordinators’ capabilities.

**Supplementary Information:**

The online version contains supplementary material available at 10.1186/s12913-024-10720-x.

**Table:** 

**Contributions to the literature**
1. The qualitative exploration of coordinators’ experience provides important information for effective institutionalization.
2. The transitional care model as a project requires cooperative relationships, a common computing platform to share patient information between institutions, and institutional assistance on patient needs and support coordinators’ capabilities.
3. It can be established as part of the general healthcare system by supplementing the limitations and developing capable factors that appeared in pilot programs or separate projects.

## Background

The transitional period is considered the most vulnerable for geriatrics or patients with chronic diseases within the healthcare system [1, 2]. It involves moving a patient undergoing treatment or care for a disease from one facility or hospital to another facility or living facility, or transfers of the medical staff or health service environment [3, 4]. Transitional care service (TCS) is an integrated service designed to ensure coordination and continuity of patients’ healthcare during the transitional period [5]. From admission to the hospital, a discharge plan is established based on the patient’s in-depth assessment and through information sharing between the patient, family, medical staff, and community connections. Consequently, patients with chronic diseases and their families can manage the disease stably after discharge. In Korea, the elderly population is rapidly increasing and is predicted to account for 20.6% of the total population by 2025 [6]. Data show that 10% of inpatients are re-admitted within 30 days of discharge [7]. Therefore, the demand for TCS to reduce readmissions is expected to increase gradually. Because readmission causes problems such as increased medical expenses, inefficient use of resources, and social costs, the United States and United Kingdom are focusing on reducing readmission rates nationally [8, 9]. In the United States, more than $52.4 billion is spent annually due to readmissions [10]. Center for Medicare and Medicaid Services (CMS) has launched a Hospital Readmissions Reduction Program (HRPP) and cut annual medical insurance benefits by 1–3% [11] for hospitals with a high readmission rate regarding six diseases (myocardial infarction, chronic obstructive pulmonary disease, etc.) to reduce the negative consequences of readmission and additional medical expenses.

Since 2019, a representative transitional care program, the Community Linkage Program for Discharge Patients (CLDP), has been implemented nationwide in Korea as part of the Public Healthcare Network Plan (PHNP) centered on Accountable Care Hospitals (ACHs) [12]. ACHs are designated as public healthcare organizations and tasked with bridging healthcare disparities and improving essential healthcare in their jurisdictions. ACHs improve essential healthcare (severe medical care, community healthcare, infection and patient safety, etc.) in assigned areas (region and intermediate care areas), focusing on public health institutions to bridge the gap in essential medical care between regions (regional or local). After the ACH concept was first mentioned in 2018, the Ministry of Health and Welfare designated 12 national university hospitals across the country as Regional Accountable Care Hospitals (RACH) and 14 local medical centers as Local Accountable Care Hospitals (LACH) in 2019 to execute PHNP [13, 14]. CLDP is the priority project among the various PHNP and is to be conducted by ACHs. In 2019, five diseases (stroke, heart, respiratory, elderly fracture, and cancer) were selected as the target disease according to the regional characteristics and hospital situation. While nurses and social workers became mandatory as dedicated personnel, doctors participated through an adjunct position [15]. The program established customized care for discharged patients, linking necessary health-medical-welfare services after discharge, sharing care plans between institutions through Public Health Connected System (PHCS, a separate computer platform), and monitoring after discharge [15]. Monitoring indicators are autonomously determined by the institution. The recommended indicators include the number of cases performed, readmission rates, emergency room visits, medication and outpatient compliances, and so forth. As of January 2023, 17 RACHs and 42 ACHs have been recognized, and the target diseases have also been expanded to chronic diseases, such as Parkinson’s disease, spine-related diseases [15]. CLDP uses the budget of the Ministry of Health Welfare and local governments (provinces and cities). Although it is on a small-scale (participating institutions and target diseases), a project that uses health insurance benefit fees is performed separately under the pilot project to support the discharge of acute patients and linkage to the community, which provides customized integrated services for inpatients with cerebrovascular diseases in acute care facilities [16]. Some national university hospitals are simultaneously participating in both projects.

Transitional care can improve patients’ health and quality of life and reduce unnecessary medical use; however, its implementation is challenging because it surpasses the traditional role of hospitals. Some randomized controlled trials (RCTs) on transitional care describe factors regarding successful and failing care. These factors establish a care plan based on the patient’s preference and participation, the coordinator’s competency, consistent high-quality follow-ups, the development of intervention factors, and evaluation indicators from the patients’ and guardians’ perspectives [17–20]. Although researchers’ insights are crucial, applying the transitional care model in practice and successfully operating it requires an in-depth understanding of the difficulties and necessary factors regarding project performance recognized by coordinators participating in actual projects. The coordinator is a key medium and subject that successfully realizes the theoretical elements of transitional care in practice. Therefore, the trial and error during the implementation stage can be reduced if the coordinator is aware of the practitioners’ experience in advance. While several qualitative studies have been conducted on patients and their caregivers, only a few involved coordinators (nurses, medical social workers, etc.) performing TCS. Specifically, Leithaus et al. [21] conducted interviews with practitioners and stakeholders who provided TCS and discussed ways to improve the platform for linking information between institutions. Toscan et al. [22] argued that clarifying the roles and division of responsibilities between practitioners or medical staff in other wards can prevent TCS duplication. Lee et al. [23] conducted an in-depth interview with medical social workers who performed a TCS and indicated difficulties, such as the absence of an assessment tool to evaluate patients’ unmet needs and lack of community resources that can be linked as obstacles. Previous studies have conducted qualitative research to derive improvement measures in the systemic [21] and structural aspects [22]. However, in-depth interviews were conducted only for medical social workers who performed TCS-related projects [23], and did not include various practitioners’ perspectives.

This study aims to explore the experiences of CLDP coordinators (nurses, social workers, etc.) in RACHs/LACHs and identifies concerns, success and limitation factors, self-improvement possibilities, and points required for institutional improvement. Unlike conventional treatment, transitional care can affect people differently depending on the target, scope of intervention, institutional conditions, and social context. In this study, CLDP is a nationwide pilot operation, and its entitlement (in the national insurance service) and institutionalization have yet to be achieved. A qualitative exploration of the working team’s experience in the project operated by each implementing institution according to the local situation will provide important information for effective institutionalization in the future.

## Methods

This study was designed to gain an in-depth understanding of the facilitating and barrier factors of formally adopting and expanding a transitional care program for discharge patients that is currently in its pilot phase in Korea. To this end, this qualitative study purposively sampled hospitals that are currently running the CLDP in Korea and aimed to understand the experiences and perceptions acquired among coordinators in charge of the project.

### Participant recruitment

The study participants were coordinators (nurses and social workers) in charge of CLDP in RACHs (mainly national university hospitals) and LACHs (mainly local medical centers and general hospital-level public medical institutions) across the country. We recruited 22 coordinators from 12 RACHs and 19 practitioners from 9 LACHs as the interviewees. Among the 22 participants from 12 RACH, 12 were nurses, 9 were social workers, and 1 was a researcher (Table 1). Their work experience ranged from a minimum of 3 months to a maximum of 3 years. Among the 19 participants from 9 LACHs, there were 10 nurses and 9 social workers. Their work experience was a minimum of 5 months to a maximum of 2 years and 4 months.

**Table 1:** General characteristics of interviewees

ID	Characteristics of Institutions	Participants’ job area (gender/ work experience) (y = year, m = month)
Region_01	National University Hospital	Nurse(F/3y), Social Worker(M/3y)
Region_02	National University Hospital	Nurse(F/2y 11 m), Social Worker(F/2y 9 m)
Region_03	National University Hospital	Nurse(F/1y 3 m), Social Worker(F/8m)
Region_04	National University Hospital	Nurse(F/1y), Social Worker(F/1y 1 m), Researcher(F/2y 9 m)
Region_05	National University Hospital	Nurse(F/3m), Social Worker(F/6m)
Region_06	National University Hospital	Nurse(F/1y 9 m), Social Worker(F/3y)
Region_07	National University Hospital	Nurse(F/2y 10 m)
Region_08	National University Hospital	Nurse(F/2y), Nurse(F/1y 11 m)
Region_09	National University Hospital	Nurse(M/ 3 m), Social Worker(F/3m)
Region_10	National University Hospital	Social Worker(M/3y)
Region_11	Private University Hospital	Nurse(F/ 9 m), Social Worker(F/7m)
Region_12	National University Hospital	Nurse(F/1y 7 m)
Local_01	Local-based public hospital	Nurse(F/2y 2 m), Social Worker(F/2y 2 m)
Local_02	Local-based public hospital	Nurse(F/2y 3 m), Social Worker(F/2y 3 m)
Local_03	Local-based public hospital	Nurse(F/10m), Social Worker(F/7m)
Local_04	Local-based public hospital	Nurse(F/2y 2 m), Social Worker(F/2y 2 m)
Local_05	Local-based public hospital	Social Worker(F/2y 4 m), Nurse(F/2y 4 m)
Local_06	Local-based public hospital	Nurse(F/5m), Nurse(M/5m), Social Worker(F/5m)
Local_07	Local-based public hospital	Social Worker(F/9m), Nurse(F/2y 3 m)
Local_08	Local-based public hospital	Nurse(F/2y 3 m), Social Worker(F/2y 3 m)
Local_09	Local-based public hospital	Social Worker(F/2y 4 m), Nurse(F/2y 4 m)

For RACH, a formal research cooperation request was first sent to the team dedicated to CLDP. Then, we called the coordinators at each hospital to provide an explanation about the study and request their participation in interviews. A few hospitals were excluded during this process for indicating inability to participate in the interviews due to short experience on the job or other reasons, and interviews were conducted with coordinators who agreed to participate. We sent the study information sheet and consent form to those who provided a verbal consent, and we obtained their signatures after providing an explanation once again during the Zoom interview. The main interviewer was a member of a dedicated team at one RACH, which facilitated cooperation with other teams. Given the large number of LACH available, we first selected those that would be more willing to participate in the interview and contacted them over the phone to provide an explanation about the study and request interview participation. Then, we sent official request letters to the consenting facilities and scheduled the interviews. Other hospitals were recruited through snowball sampling, and consents were obtained through the same method.

### Data collection

Interviews were conducted between March and April 2022 for RACH coordinators and between July and August 2022 for LACH coordinators. Interviews were conducted online (via Zoom), by two interviewers. There were 1–3 practitioners in charge of the project for each institution, including nurses, social workers, and researchers, depending on the institution, who participated in the interview together. The interview was conducted based on the semi-structured questionnaire that we developed for this study (Table 2). Also the interview content has not been published elsewhere. The interview questions were developed with discussion with YP and SMJ focusing on eliciting responses that reveal the facilitators and barriers of the CLDP (Table 2). YP, a manager of CLDP at one of a RACH, brings two years of experience as a physician in the team. YP has actively participated in weekly multidisciplinary meetings with coordinators, engaging in extensive communication to support the project. The questions were designed based on these experiences, beginning with open-ended inquiries about each hospital’s unique protocols and environments to delve deeper into the challenges faced by each hospital, and their efforts and limitations in improving these processes so as to uncover answers to the study questions. Details are attached to Additional File 1.

**Table 2:** Semi-structured interview questions

Question area	Detailed questions
Business progress and results	Please feel free to explain the process and intervention elements of the CLDP that your institution has carried out thus far.
	How were the elements and processes of intervention developed?
Difficulties associated with conducting business	At your institution, which process has not gone as planned (guidelines) thus far in your experience of CLDP?
	What could be the reason?
	What has been the toughest thing for you in performing the project?
Self-evaluation of business effectiveness	What were the monitoring results and CLDP performance performed by your institution thus far?
	What do you think about the factors causing the favorable and unfavorable areas from your self-evaluation?
	Do you have any plans to improve the unfavorable areas?
Efforts to improve business	Please introduce any basic research that your institution has conducted that is related to the project.
	Apart from the guidelines, what do you think could be necessary as interventions to meet patients’ needs and achieve favorable results?

The interview lasted for 60–90 min. Further interviews were requested and conducted or confirmed by phone or e-mail when additional questions or confirmation of meaning were needed during the analysis process. Based on informed consent, the interview process was recorded. The interviewer conducted a debriefing meeting and wrote on-site notes after the interview. Transcripts of interview recordings and field notes were analyzed.

### Data analysis

Thematic analysis was used to analyze the data. This method systematically identifies and organizes patterns in the meaning (themes) of collected data and for gaining insights. Thematic analysis is a way of analyzing qualitative data that has the advantage of being accessible and flexible [24].

This study performed an inductive approach to derive themes from data and proceeded with the 6-phase approach presented by Braun and Clarke [25]: (1) familiarizing oneself with the data, (2) generating initial codes, (3) combining codes for themes, (4) reviewing potential themes, (5) defining and naming themes, and (6) producing the report. To ensure the rigor of this qualitative study, efforts were made to meet the four criteria set by Lincoln and Guba [26]. In terms of credibility, the main interviewer is a manager of the dedicated team at a RACH and has participated in meetings and observed the project’s progress for over 2 years. This helped in building trust with the teams at other hospitals based on shared experience in the project’s management. To establish dependability, the study process was detailed meticulously. We conducted data analysis strictly in accordance with the process outlined by Braun and Clarke [25], and a consensus was reached for the results through a process involving repeated reading of the data and sharing and gaining feedback within the research team on the derived themes. For confirmability, two interviewers participated, and debriefing sessions and individual field notes were used to minimize subjective interpretation in the analysis. Preliminary analysis results were presented to various related groups for feedback. To achieve transferrability, detailed descriptions of participants’ general characteristics and data collection procedures were provided, enabling readers to understand the context and applicability of the study findings.

All methods were performed in accordance with the declaration of Helsinki. The study was approved by the Kangwon National University Hospital IRB (registration number: A-2021-10-011-004), and informed consent was obtained from each participant.

## Results

### Theme 1: formation and maintenance of cooperative relationships

The success or failure of the CLDP is determined by how well cooperation exists between different departments within the hospital, between related departments, and between institutions that can link the services that patients need outside the hospital after discharge. This theme served as both a facilitator and barrier depending on the internal and external context of the hospital.

### Degree of participation of medical staff (doctors)

Institutions reported that TCS progress successfully, especially with doctors actively cooperating with patient referrals, establishing discharge plans, and participating in multidisciplinary meetings. Physicians’ collaboration is thus key to recruiting patients and developing discharge plans; however, in the current hospital system, this task is not mandatory and is ordinarily assigned to physicians. Therefore, even if the project team provides incentives to request additional work, improving the TCS quality is difficult in cases where doctors seldom set aside separate time due to the nature of the specialization or inadequate cooperation because of individual tendencies.

*It is tough to get the doctors together when we establish a discharge plan. However, in our case, it’s working well. The project is progressing thanks to the active participation of doctors…(Local_06)*

*We have a concept like a case conference with cases that have been referred to services through our program. When I request discussion of four doctors for some complex cases, they deliver well. (Region_07)*

*I am requesting to establish a care plan for the doctoral sector, but it is not working well, and doctors are annoyed. That is why we are not doing well in expanding the medical specialties. (Region_10)*

### Existence of a cooperative structure with related institutions

Cooperation with related organizations, which is how well different organizations can connect and collaborate to meet the patient’s changing needs, is also crucial. Participants responded that connecting discharged patients with institutions having an official cooperation system, such as a memorandum of understanding (MOU), is easier.

*We are enrolled in PHCS with 10–12 organizations that we have MOUs with. When the institution’s ID is held, the patient can be referred and receive feedback. However, it is a bit difficult for others that are not cooperative institutions. For some institutions, having 1–2 cases per year, cooperating through PHCS becomes difficult. They tend to respond more smoothly to previously registered institutions. (Region_02)*

### Coordination between other departments and projects within the institution

Institutions that are well-coordinated with other departments with similar processes help each other by sharing subjects. If the coordination is inadequate, it becomes difficult for the project to proceed. This includes departments that have mainly consulted patients with financial difficulties and linked cost support and similar projects that are provided through other government departments. This problem also arises because the current project is not yet included or institutionalized in health insurance and is functioning with a separate budget.

*Medical benefit recipients or low-income patients already receive intervention and management through other support teams (i.e., the hospital’s existing social work team), and (omitted) I think it would be a bit difficult for the patient to have overlapping consultations with nursing and welfare from this team and welfare from that team. That’s why, if possible, we try not to intervene with patients assigned by other teams… (Local_01)*

### Theme 2: communication and information sharing system for patient care

CLDP establishes a multidisciplinary, cross-professional, team-based care plan within the hospital and provides continuous services by transferring information outside the hospital as transitional care. Therefore, the amount of patient information shared inside and outside the hospital and communication between practitioners might greatly influence the success or failure of a project. In this theme, computer systems were a facilitator in multidisciplinary communication within the hospital, but a barrier in communications with other external organizations. On the other hand, unofficial messenger served as a facilitator, showing different roles of computerized systems depending on context.

### Communication between multi-disciplinary team within hospital

The computer systems within the hospital promote information sharing and communication. In most hospitals, the electronic medical report (EMR) does not include a referral system for multidisciplinary communication and a linkage program for discharging patients in hospitals because Korean hospitals have not yet institutionalized transitional care services. An active coordinator uses phone calls and messages to operate, which consumes individual energy and has limitations regarding individual capabilities. Therefore, computer programs were developed and used in-house in a few cases.

*The first time, we did not request it electronically through EMR. Therefore, I sent and received text messages and referred the patients. I visited the patients and explained. However, I asked doctors to develop a program with the computer team. When the request screen appears in the window now, click this on our side, and it will be commissioned (Region_03)*

### Platform for sharing patient information with community organizations after discharge and linking services

PHCS is a computer platform operated by the National Medical Center that helps CLDP of ACH share nursing, medical, and social welfare evaluation information and care plans for discharging patients to other institutions. Although this patient information-sharing process is safe, accessing and causing additional loading is cumbersome because official authentication to access a separate platform can obstruct cooperation.

*It becomes too cumbersome and difficult to use the network (*PHCS). *After at least five cases a month, if the system continues to connect the patient, it asks to designate a person in charge to certify and proceed. However, the number of cases is not much for us. I wonder if one patient is linked to a base once every two or three months. Therefore, it is also troublesome on the other side. In case, I connect like this, the person in charge may get changed. (Region_01)*

*Originally, they processed in their usual way and went back and forth with one sheet to finish it. However, as we give this vast amount of data (through the network) to check and ask for an answer, it goes far (omitted). They said something like, “I cannot give it to you or take care of all of them because there are too many welfare centers.” (Local_02)*

Regarding linking services, some local governments have officially established integrated care headquarters to create separate entry points that connect various community care services. However, although informally, the linkage is easier when an environment is established to check and connect the necessary services for discharging patients in real-time through a messenger channel (KakaoTalk) created by the other local government.

*KakaoTalk group chat room is created by E[pseudonyms] county, having 200 people. Civilian heads of the E county and all people related to social welfare are included in this chat room. (Omitted) Because this group chat room is created by the E county, a lot of interest exists within the E county itself. (Omitted). If a case occurs or we have a request, we post it in the group chat room. When we go to a home visit and say that we need such and such things (upload a group chat), it is received right here. The person in charge connects the necessary resources and informs the results. I think the service is rather good. (Local_05)*

### Theme 3: Interaction among program, regional, and individual capacities

CLDP provides the basic level of guidelines, framework, and high autonomy for each area and institution to establish specific action plans. Therefore, programs are tailored to each area and hospital, but the long-term direction of the project becomes challenging. When guidelines are ambiguous or absent, the success or failure of a project depends more on the individual coordinator’s capacity and the area. In addition, since CLDP assigns roles to hospitals (ACHs) and coordinators only, it is difficult to overcome the community service linkage outcome with the existing service infrastructure and capacity of the area. Despite the diversity of regions and hospital workforce competencies, the loosely structured nature of this nationwide project often was a barrier for coordinators.

### Confusion due to loose guidelines and lack of direction

Coordinators who participated in the interview felt rewarded from individual cases thinking that they were helping patients but were struggling to ensure that this project was moving in a direction that would ultimately benefit patients. Institutions mainly monitored performance based on activity indicators related to linkage (not on outcome indicators through linkage) and recognized that current intervention levels are insufficient to improve common monitoring indicators, such as readmission rates. In addition, although common indicators are recommended, the intervention content and monitoring indicators are determined by the ACH’s discretion. Therefore, the coordinator tends to be confused about which direction to go in.

*In the case of our hospital, I think a part contributes to solving the blind area to some extent, as resources are linked anyway through the project. Therefore, when staying at home, you will not know this. You can come to the hospital and be informed of the resources. When I think about collaborative methods, these things seem to have a positive effect. (Local_06)*

*First, I think that this project is inadequate to control the readmission rate. I wonder whether using the readmission rate as an indicator through this project is correct. (Region_10)*

### Variation according to individual competency

Apart from program organization, the coordinators have more significant roles in TCS. Identifying patient needs, embracing the opinions of different professions, and discovering and linking services provided by different organizations will depend on how actively the coordinator coordinates and communicates. However, improving the quality of work sufficiently is difficult in institutions where the person in charge is changed frequently or has insufficient experience. The project guideline, which is that the coordinator in charge must be a full-time employee, is not mandatory but recommended, and the coordinator’s capacity changes according to the institutional circumstances because it runs with government subsidies.

*We had a multidisciplinary team conference on case management and provided the following feedback regarding the content of the feedback, what the professors were curious about, or if this part would be supplemented or corrected, received feedback from the institution, and replied to the institution. Further, we asked the institution, “Can you give me this part? I think I need a little bit of this part.” And the institution took care of what they could do for us. (Region_07)*

*By the time the project is well established and the coordinator is comfortable, their contract ends and they leave. It is good if a new person can take over the job, but when a new coordinator comes on board a few months after their predecessor leaves, someone has to know about the project. When these persons rejoin, they should be restored, taught, and perform something again, and this vicious cycle continues. A contract worker can leave whenever desired before a new person joins. (Region_07)*

### Gaps in care services that can be linked in the community

The inpatient residence may vary beyond the city and county where the hospital is located. However, Korea’s welfare system is based on city, county, and district rather than the area/region or middle medical care area designated in this project. Therefore, patients living in different cities and counties receive the same TCS program at the hospital. However, to receive social services in the community after discharge, patients are dependent on the infrastructure and services of the residing city or county. Many services can be linked in large, highly populated cities, but are scarce in counties with a small population. Inequality in linked services, even for the same project target, is also noticed. In addition, the eligibility for services linked to the geriatrics or the economically vulnerable is limited in several cases. Community care case management is not universally equipped, and most patients who do not meet the (mostly financially) qualifications have to purchase services by themselves. The limitation is that there are few community services that coordinators can connect with.

*Some patients can receive benefits, but other patients do not receive the same, depending on where they live. Therefore, for these cases, how can we solve this problem? (Region_03)*

*If I meet a person with difficulties, I try to connect at least once (omission). Most patients are recipients of basic livelihood security or are in the next upper-class level and are already receiving services. They could not receive additional services because the criteria are not met. For general patients, it is too difficult to connect with support because their income level is not met. (Local_01)*

## Discussion

This study explored the factors that facilitate and obstruct the transitional care program by revealing the experiences through interviews with coordinators of the CLDP, which has been operating as part of the PHNP in Korea since 2019.

Although the facilitating and barrier factors experienced by practitioners were expected to be different because of the size of hospitals and severity of patients in RACHs and regional LACHs (RACHs are university hospitals with 600–1000 beds, and LACHs are general hospitals with 100–400 beds). However, no additional themes were emerged during the process of coding and deriving themes from the LACHs interviews, which converged on the common themes found in the RACH. The characteristics or size of the hospital did not affect the implementation-related factors because the main project content was the assessment by coordinators and linkage with community resources rather than patient’s treatment in the hospital. While not presented as results due to the focus of this study, we did see differences in the range of target patients. Contrary to project protocols that designate inpatients with specific illnesses to support in-depth evaluation and disease management, LACHs preferred a universal management protocol to manage general geriatrics or patients with chronic diseases without limiting diseases. When LACHs designate a specific disease, the number of target inpatients is not large. Rather, the proportion of elderly patients with multiple chronic diseases is high due to the characteristics of the area.

Partnership inside and outside the hospital were cited as major facilitating and barrier factors to project implementation, as it is essential in transitional care to have multidisciplinary care for patients and cooperation with the community after discharge (Theme 1). Specifically, the doctor’s cooperation greatly affects both the simpler and more cumbersome areas [21, 27, 28]. Coordinators in transitional care are mainly nurses or social workers, and compensating for patients’ medical problems without doctors’ cooperation is not possible. The imbalance in the power relationship between doctors and other staff within the hospital also complicates the coordinator’s cooperation without the individual will and doctors’ initiative [21, 27]. Moreover, in the case of existing doctors or nurses, cooperation becomes difficult because of the additional effort rather than the original workload in the hospital [21]. Doctors can actively participate through regular multidisciplinary meetings, but it is not an essential intervention element in the CLDP project. Therefore, only a few hospitals held these meetings, which is helpful for patient care, and similarly noted in other studies [29, 30]. Several hospitals observed that a formal framework such as MOU, which is prepared at the organizational level, helps cooperate with external organizations [31] and clarifies the role of cooperation. In this study, an informal communication method led by the government shares services and exchanges links by forming a linkage channel (KakaoTalk chatroom) between related organizations centered on the county. Conflicts arising from overlapping departments or programs with similar roles were mentioned as obstacles to cooperation within the hospital. A study by Lutz et al. [31] stated protocol modification while integrating a similar existing program when running a newly introduced program as being a problem. This study revealed inefficiency and insufficient cooperation when similar programs were operated separately within one hospital due to the lack of a flexible protocol and separate CLDP form.

Sharing care plan information within the multidisciplinary team and ensuring a system that supports continuous care during transitions in the environment and care providers were found to be crucial for addressing patients’ complex needs (Theme 2). In this regard, there was a case in which a tailored computer system specific for the hospital was developed by convincing the hospital’s IT team, and the developed system facilitated identification of patients and multidisciplinary communications. In contrast, the computer systems for information sharing between hospitals and other institutions were mainly cited as an obstacle. The CLDP can safely share patient information between institutions through a separately developed computer platform called PHCS. As this system does not automatically link with the hospital’s computer program, it is necessary to fill out and input a separate form and secure access through an official certificate to check patient information from an external institution and the requested patient’s information. This additional load outside of work eventually hinders cooperation, suggesting that accessibility of hospitals and local practitioners and integration with existing systems must be considered when developing a patient information-sharing platform. For the computer system to function as a facilitator, an integrated system that provides easy access to patient information inside and outside the hospital would be necessary.

While loose and ambiguous project framework and guidelines could provide autonomy, they generally could not prevent the risk for regional disparities and caused confusion regarding the specifics and direction of work for coordinators (Theme 3). Practitioners in this study expected that this project could be helpful to patients because it provided disease education to patients and connected services directly. However, achieving sufficient long-term results to reduce patients’ readmission with the limited time and resources by using few coordinators is doubtful. Therefore, some hospitals have even planned to visit patients directly to overcome unclear feedback through affiliated institutions. This project has further limitations. Fundamentally, evaluating the project’s effectiveness in the central department (the department that manages this project) is mainly focused on quantitative activity performance. The evaluation of actual improvement in patients’ lives is left to the discretion of the hospitals.

The coordinator’s initiative and capability operate as major factors during the ambiguity of the project guidelines. Fakha et al.’s [32] study was consistent with the assertion that if the organizational factor was a main bottleneck, an individual factor, such as a practitioner’s strong will and commitment, is the facilitator. Even in programs with the same interventional elements, a significant difference was found in quality depending on how actively the coordinator understands patients’ needs, shares feedback among medical staff, and searches for and connects with community services. Fakha et al. [32] suggested that, in addition to the activeness of practitioners in charge, engaging active advocates of the program is significant, both inside and outside the organization. In the study by Sun et al. [28], mutual trust increases with quick responses and immediate feedback on the needs of providers, highlighting the importance of the coordinator’s role. Simultaneously, the rotation of practitioners was identified as an obstacle [29, 30]. In our case, when the coordinator was assigned as a non-regular worker, the capacity was not accumulated, but was always reset because it was changed periodically. Agerholm et al. [27] revealed that transitional care could be successful when practitioners cross the boundaries of each other’s tasks and become more active despite the additional load. However, it is necessary not to be fundamentally influenced by individual capabilities when systematizing the guidelines. Because individual commitment inevitably leads to burnout, human and financial support for the transitional care coordinator and educational support to strengthen competence are necessary in the long term.

Patients require a proper connection with the health and welfare services provided by the community to suit their needs of residing at home when not fully recovered after discharge. This connection enables sufficient recovery, living in one’s familiar home, preventing unnecessary re-hospitalization due to aggravation of the disease or failure of care, and life satisfaction. However, coordinators in this study regretted that despite comprehensively evaluating the patient’s needs, proper health and welfare services could not be provided due to inadequate services linked to the community or difficult standards (age, socioeconomic level, etc.). Because the social service system in Korea is established based on cities, counties, and districts, gaps exist in the social welfare infrastructure and services provided depending on the region [33]. Although the coordinator at the hospital identifies patients from various regions with the same needs, the patients can receive unequal services after discharge depending on their place of residence, inevitably leading to unequal health outcomes. Lutz et al. [31] cited medical staff’s lack of familiarity with community resources as an obstacle to transitional care. Sun et al. [28] identified that hospitals and community organizations that operate independently with different systems make linkage difficult [29, 30]. Therefore, expanding social services to provide sufficient care at home is essential for patients in the recovery period after discharge, even if they are not elderly or vulnerable. Moreover, medical, welfare, and health services, which operate in different systems, must be integrated at the macrosystem level (financial or legal).

All RACHs, except those that refused to participate due to lack of experience shortly after their designation, were investigated. This is expected to cover most of the facilitating and barrier factors to implementation that are experienced by public medical institutions at the level of university hospitals. Compared to the initial expectation that the factors of LACHs differ from that of RACHs, similar factors were derived, and saturation was eventually confirmed and terminated. However, generalizing the practical aspects of small- and medium-sized hospitals was difficult because limited LACHs were investigated. Other themes may remain with special conditions and local contexts. Special contexts may also exist depending on the disease due to the nature of transitional care. Although each medical institution targeted different diseases, they remained unspecified. Additional research focusing on specific diseases, such as cardiovascular, cerebrovascular, and respiratory diseases, will be needed.

## Conclusion

This study explored and derived the facilitating and barrier factors for the model’s execution through coordinators’ experience in cases of transitional care projects conducted nationwide in a society where community care issues are increasing.

Multidisciplinary collaboration within the hospital and between community institutions was identified as the key factor determining the success or failure of meeting complex needs of patients. Various incentives for motivation should be ruminated upon while considering the additional workload of doctors, nurses, and social workers who perform existing roles in hospitals. Since the launch of the CLDP, similar transitional projects led by local governments and other central organizations are emerging. Similar institutional devices can enable cooperation between projects centered on patients and prevent each transactional project from being managed separately and operating inefficiently.

A computing platform should be integrated from hospitals into local communities and primary care institutions according to the flow of patients to facilitate communication and information-sharing. Practitioners should be supported and empowered to improve project quality. In addition to hospitals, personnel who are capable of coordinating patients at each point of their movement are also essential.

All institutional improvements must be patient-centered. Patterns of patients’ changing needs must be analyzed from their perspective to develop support personnel and services at the appropriate points. Short-, mid-, and long-term performance indicators focusing on patients’ quality of life should be established. This will ensure that coordinators remain focused and self-efficacy is increased, which will contribute to their patients’ wellbeing.

In this study, coordinators’ experiences revealed the interface between patients and institutions. In future, this is expected to be established as part of the general healthcare system by supplementing the limitations and developing capable factors that appeared in pilot programs or separate projects.

### Electronic supplementary material

Below is the link to the electronic supplementary material.

Supplementary Material 1

## Data Availability

The data that support the findings of this study are available from the corresponding author upon reasonable request.

## Abbreviations

RACH: Regional Accountable Care Hospital
LACH: Local Accountable Care Hospital
PHNP: Public Healthcare Network Plan
CLDP: Community Linkage Program for Discharge Patients
PHCS: Public Health Connected System
