# Supplementary material for: Facilitating and barrier factors to the implementation of a transitional care program: a qualitative study of hospital coordinators in South Korea
Source: BMC Health Serv Res. 2024 Feb 23;24:240. doi: 10.1186/s12913-024-10720-x (PMC10893592; doi:10.1186/s12913-024-10720-x)
Supplement: Supplementary file 1 — Supplementary Material 1 [file 12913_2024_10720_MOESM1_ESM.docx]

Interview Questionnaire for Practitioners to Develop a Patient-Centered Transition Care Management Program

- Process and Results of Community Linkage Program for Discharge Patients(CLDP)
  - What are the target diseases for the CLDP conducted in your institution so far?
  - Please freely describe the procedures and intervention elements of the CLDP for each disease.
  - How were the procedures and intervention elements developed initially?
  - (If you have experience in respiratory disease projects) Were there any particular considerations or challenges in implementing the program?
- Research Experience Related to CLDP
  - What kind of basic research has your institution conducted so far regarding CLDP?
  - To what extent do you think the results of this basic research have been incorporated into the current programs?
  - If there are parts that have not been incorporated, what is the reason?
- Challenges in Implementing CLDP
  - In your institution's experience implementing CLDP so far, what parts did not proceed as planned (according to guidelines)?
  - What do you think were the reasons for this?
  - What is the most challenging part of implementing the program?
- Experience in Self-Evaluation of the Effectiveness of CLDP
  - What have been the monitoring results and achievements of CLDP carried out so far?
  - If there are parts that have or have not worked well in your self-evaluation, what factors do you think contributed to this?
  - What are your plans to improve the parts that have not worked well, if there are any?
- Limitations in the Guidelines and Budget for CLDP, and Requirements for Improvement
  - Do you believe that the guidelines and budget currently provided by the Ministry of Health and Welfare are sufficient for your institution to implement the most effective interventions?
  - What are shortcomings in the existing guidelines, if any?
  - Apart from the guidelines, what additional intervention elements do you think are necessary to meet patient needs and achieve excellent outcomes?
  - What improvements do you think should be made to the current guidelines or budget to meet these needs?

- 1 -
